# 571. Safety and Immunogenicity of Coadministered Bivalent BNT162b2 COVID-19 Vaccine and Bivalent RSVpreF Respiratory Syncytial Virus Vaccine With and Without Quadrivalent Influenza Vaccine in Adults ≥ 65 Years of Age

**DOI:** 10.1093/ofid/ofae631.172

**Published:** 2025-01-29

**Authors:** Joel Neutel, Rahsan Erdem, Qin Jiang, Kevin Cannon, Helen Stacey, Ryan Newton, Emily A Gomme, Wen Li, Federico Mensa, Ozlem Tureci, Ugur Sahin, Kena A Swanson, Iona Munjal, David Cooper, Kenneth Koury, Annaliesa S Anderson, Alejandra C Gurtman, Nicholas Kitchin

**Affiliations:** Orange County Research Center, Santa Ana, California; Vaccine Research and Development, Pfizer Inc, North Potomac , MD; Pfizer, Collegeville, Pennsylvania; Accellacare, Wilmington, North Carolina; Diablo Clinical Research, Walnut Creek, California; Vaccine Research and Development, Pfizer Ltd, Marlow, England, United Kingdom; Pfizer, Collegeville, Pennsylvania; Pfizer Vaccine Clinical Research, Collegeville, Pennsylvania; BioNTech, Mainz, Rheinland-Pfalz, Germany; BioNTech, Mainz, Rheinland-Pfalz, Germany; BioNTech, Mainz, Rheinland-Pfalz, Germany; Pfizer, Collegeville, Pennsylvania; Pfizer Inc; Pfizer, Collegeville, Pennsylvania; Pfizer, Collegeville, Pennsylvania; Pfizer, Collegeville, Pennsylvania; Pfizer, Collegeville, Pennsylvania; Pfizer Inc

## Abstract

**Background:**

Older adults are vulnerable to both severe COVID-19 and RSV illness. Coadministration of RSV and COVID-19 vaccines could reduce healthcare visits for this population and potentially improve vaccine uptake. This study evaluated safety, tolerability, and immunogenicity of RSV and COVID-19 vaccines given together with or without seasonal quadrivalent influenza vaccine (QIV) as part of a larger clinical study looking at combination vaccines.

**Figure:**
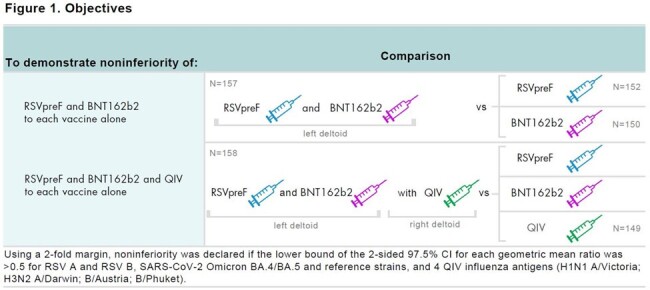

**Methods:**

This phase 1/2 randomized study (NCT05886777) included healthy ≥ 65-year-olds who had received ≥ 3 previous mRNA COVID-19 vaccine doses (the most recent a bivalent vaccine given ≥ 150 days before study vaccine). In this study part, participants received coadministered RSVpreF and bivalent BA.4/BA.5-adapted BNT162b2 vaccine (RSVpreF/BNT162b2) with QIV or placebo; RSVpreF; BNT162b2; or QIV. Immunogenicity objectives included demonstration of noninferiority of neutralizing antibody titers elicited by RSVpreF/BNT162b2 compared to either vaccine administered alone, and by RSVpreF/BNT162b2 coadministered with QIV compared to RSVpreF, BNT162b2, and QIV alone (Figure 1).

**Figure:**
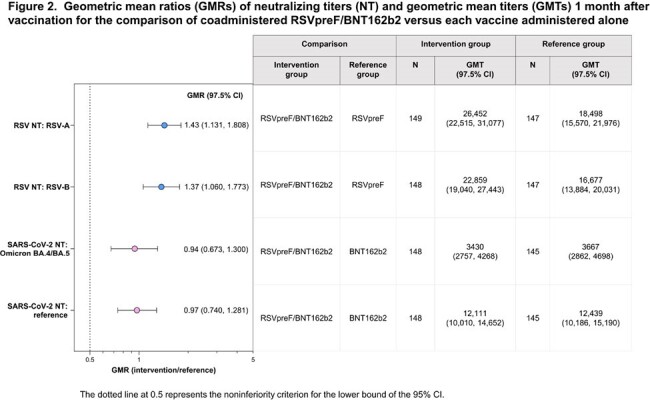

**Results:**

Of 766 participants in this analysis (149–158 per group), 46% were male, 89% White, 9% Hispanic/Latino. Median age was 71 (range 65–90) years. Based on the prespecified 2-fold margin, immune response noninferiority was demonstrated for RSVpreF/BNT162b2 for 2 RSV antigens and 2 SARS-CoV-2 strains versus RSVpreF or BNT162b2 alone (Figure 2). Noninferiority was also demonstrated for all 8 antigens (2 RSV; 2 SARS-CoV-2; 4 influenza) for immune responses elicited by RSVpreF/BNT162b2 administered with QIV versus those with RSVpreF, BNT162b2, or QIV alone (Figure 3). RSVpreF/BNT162b2 was well tolerated with mostly mild/moderate reactogenicity with rates generally similar across groups (Figure 4). Adverse events (AEs) were infrequent, mostly mild/moderate, and occurred at similar frequencies across groups. No AEs led to study withdrawal, and no serious AEs were considered vaccine related.

**Figure:**
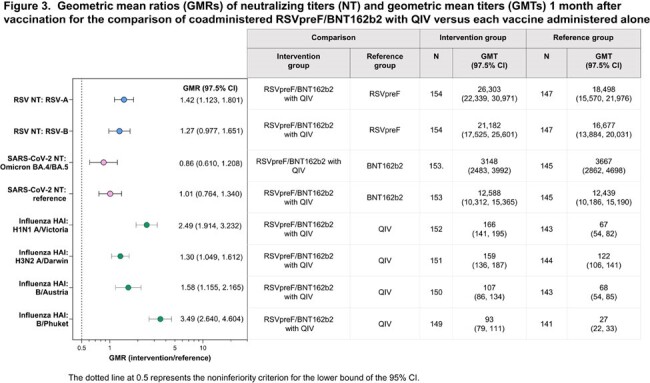

**Conclusion:**

In ≥ 65-year-olds, RSVpreF/BNT162b2 was safe and well tolerated, including when coadministered with QIV. The data support that RSVpreF and BNT162b2 can be administered concomitantly, which may support improved uptake of both vaccines.

Funding: Pfizer Inc.

**Figure:**
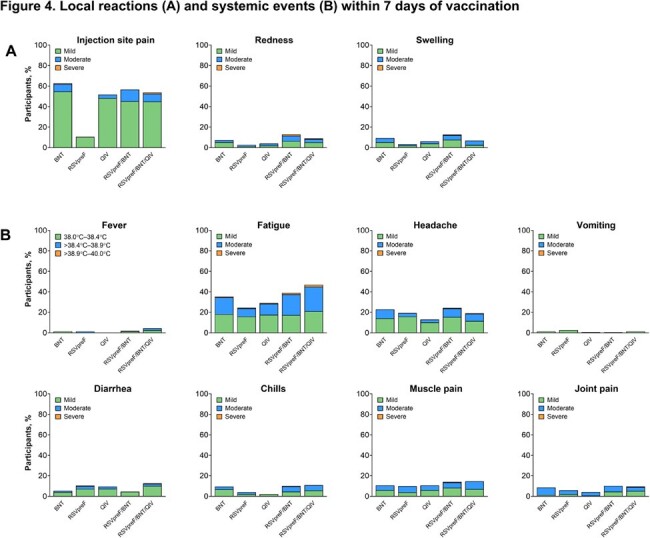

**Disclosures:**

**Joel Neutel, MD**, Pfizer Inc: Employee **Rahsan Erdem, MD**, Pfizer Inc: Employee|Pfizer Inc: Stocks/Bonds (Private Company) **Qin Jiang, PhD**, Pfizer: Salary|Pfizer: Stocks/Bonds (Public Company) **Ryan Newton, BSC/MBA**, Pfizer Inc: Employee|Pfizer Inc: Stocks/Bonds (Private Company) **Emily A. Gomme, Ph.D.**, Pfizer Inc: Employee|Pfizer Inc: Stocks/Bonds (Private Company) **Federico Mensa, MD**, BioNTech: Employee|BioNTech: Stocks/Bonds (Public Company) **Ozlem Tureci, MD**, BioNTech: Employee|BioNTech: Stocks/Bonds (Public Company) **Ugur Sahin, MD**, BioNTech: Employee|BioNTech: Stocks/Bonds (Public Company) **Kena A. Swanson, Ph.D.**, Pfizer: Employee of Pfizer|Pfizer: Stocks/Bonds (Public Company) **Iona Munjal, MD**, Pfizer: Salaried employee|Pfizer: Stocks/Bonds (Public Company) **David Cooper, PhD**, Pfizer, Inc.: Employee|Pfizer, Inc.: Stocks/Bonds (Public Company) **Kenneth Koury, PhD**, Pfizer Inc: Employee|Pfizer Inc: Stocks/Bonds (Public Company) **Annaliesa S. Anderson, PhD**, Pfizer, Inc.: Employee|Pfizer, Inc.: Stocks/Bonds (Public Company) **Alejandra C. Gurtman, M.D.**, Pfizer, Inc.: Employee|Pfizer, Inc.: Stocks/Bonds (Public Company) **Nicholas Kitchin, MD**, Pfizer Inc: Employee|Pfizer Inc: Stocks/Bonds (Public Company)

